# Axis I and II disorders as long-term predictors of mental distress: a six-year prospective follow-up of substance-dependent patients

**DOI:** 10.1186/1471-244X-7-29

**Published:** 2007-06-26

**Authors:** Kjell Bakken, Anne Signe Landheim, Per Vaglum

**Affiliations:** 1 Centre for Addiction Issues, Department for Substance Abuse, Innlandet Hospital Trust, Norway.; 2 Department of Behavioural Sciences in Medicine, Faculty of Medicine, University of Oslo, Norway.

## Abstract

**Background:**

A high prevalence of lifetime psychiatric disorders among help-seeking substance abusers has been clearly established. However, the long-term course of psychiatric disorders and mental distress among help-seeking substance abusers is still unclear. The aim of this research was to examine the course of mental distress using a six-year follow-up study of treatment-seeking substance-dependent patients, and to explore whether lifetime Axis I and II disorders measured at admission predict the level of mental distress at follow-up, when age, sex, and substance-use variables measured both at baseline and at follow-up are controlled for.

**Methods:**

A consecutive sample of substance dependent in- and outpatients (n = 287) from two counties of Norway were assessed at baseline (T1) with the Composite International Diagnostic Interview (Axis I), Millon's Clinical Multiaxial Inventory (Axis II), and the Hopkins Symptom Checklist (HSCL-25 (mental distress)). At follow-up (T2), 48% (137/287 subjects, 29% women) were assessed with the HSCL-25, the Alcohol Use Disorders Identification Test, and the Drug Use Disorders Identification Test.

**Results:**

The stability of mental distress is a main finding and the level of mental distress remained high after six years, but was significantly lower among abstainers at T2, especially among female abstainers. Both the number of and specific lifetime Axis I disorders (social anxiety disorder, generalized anxiety disorder, and somatization disorder), the number of and specific Axis II disorders (anxious and impulsive personality disorders), and the severity of substance-use disorder at the index admission were all independent predictors of a high level of mental distress at follow-up, even when we controlled for age, sex, and substance use at follow-up.

**Conclusion:**

These results underscore the importance of diagnosing and treating both substance-use disorder and non-substance-use disorder Axis I and Axis II disorders in the same programme.

## Background

A high prevalence of lifetime psychiatric disorders among help-seeking substance abusers has been clearly established [1-3]. Comorbidity is found to contribute to readmission for both the substance use disorder (SUD) and the mental disorder, and concerning treatment outcome conflicting results have been reported (3) However, the long-term course of psychiatric disorders and mental distress (symptoms of anxiety and depression) among help-seeking substance abusers is still unclear. The literature is somewhat contradictory concerning the course of mental distress in substance-abusing patients. A *decrease *in mental distress over time has been reported in patients dependent on opiates [4], cocaine [5,6], and alcohol [7-11], as well as in mixed samples [12]. In contrast, other studies have reported *stability *in cocaine-dependent patients [13], in alcohol-dependent patients [14,15], and in mixed samples [16]. Some studies with more than one follow-up have shown a decrease from baseline to the first follow-up and then stability [17]. These contradictory findings may be due to differences in sampling, the prevalence of sober patients, the duration of follow-up intervals, and the methods used to measure mental distress. Therefore, there is a clear need for prospective, long-term follow-up studies of clinically representative samples that are evaluated with reliable and valid methods at the index admission to treatment.

Several studies found no association between the severity of baseline substance use and later mental distress. This was the case for patients dependent on alcohol [18], cocaine [13], as well as abusers of amphetamines and cocaine [19]. Other studies found a correlation between the severity of substance dependence at baseline and psychopathology at follow-up. This was observed by Carroll et al. in cocaine abusers [5], and Rounsaville et al. [20] in alcohol-dependent patients, but only among men. The reasons for these contradictory findings may again be due to differences in sampling, the method of measuring the severity of substance dependence and mental distress, and differences in the time between admission and follow-up.

Several factors have been studied as possible predictors of levels of mental distress at follow-up: psychopathology, substance use factors, and sociodemographic factors. A number of studies have found an association between the number of Axis I disorders and mental distress at follow-up [20-24]. A recent review of mainly short-term follow-up studies of substance-use disorder (SUD) patients found that Axis II disorders were often associated with poor psychiatric outcomes, such as suicidal behaviour, anxiety, and depression [25]. There is a lack of long-term outcome studies (> 5 years) focusing on the importance of Axis I and II disorders for the level of mental distress in SUD populations. It is also important to control for substance use both at index admission and at follow-up when the impact of Axis I and II disorders on the course of mental distress is explored.

Against this background, we have conducted a six-year prospective follow-up study of a consecutive sample of extensively assessed treatment-seeking substance-dependent patients, from inpatient and outpatient facilities in two counties in Norway. Our research questions were:

1. What is the level of, and change in mental distress six years after admission in the total sample and in different subgroups (sex, age at onset of SUD, main substance of abuse, and Axis I and II disorders)?

2. Do lifetime Axis I and II disorders measured at admission predict the level of mental distress at follow-up, when age, sex, and substance-use variables measured at both baseline and follow-up are controlled for?

## Methods

### Sampling

A consecutive sample (n = 287) (70% men, mean age = 38.6 ± 11.3 years) of DSM-IV substance-dependent patients (156 alcohol dependent and 131 poly-substance dependent) from three outpatient (n = 157) and six inpatient (n = 130) public facilities in two Norwegian counties were recruited from September 1997 to November 1998. In the six inpatient units, there were two therapeutic communities, one shelter, one short-term unit (six weeks), and two long-term units (between 3 and 18 months). Two of the inpatient units were mainly for males, and one of the units mainly for females. The therapeutic communities offered treatment for young drug addicts. The programs had abstinence and rehabilitation as their primary goals and they offered both individual and group therapy. Few of the programs assessed and treated psychiatric disorders. The treatment in most programmes was not based on a specific ideology or philosophy. The three outpatients units offered mainly individual therapy, and the main goals for treatment were abstinence and rehabilitation.

The inclusion criterion for patients from the outpatient facilities were at least three consultations, whereas in the inpatient units, the patients had to stay at least for two weeks to be included in the study. A total of 287 patients from 690 consecutive subjects, who fulfilled the inclusion criteria, were recruited to our index admission sample. Thus, the participation rate was 42% (287/690). Those who were not recruited either refused to participate, left treatment prematurely, or most often, the clinicians did not properly present the study proposal to the patient. Those who were and those who were not recruited did not differ significantly with regard to sociodemographic variables or substance-use variables, except for age. Our sample was somewhat older than the non-participants (38.6 years vs. 35.6 years, respectively, P < 0.001). Compared with a national sample (n = 5000) drawn from the entire Norwegian treatment-seeking population [26], our sample was somewhat older (23% vs. 36%, respectively, were younger than 30 years, P < 0.001) and more frequently abused alcohol (63% vs. 50%, respectively, P < 0.001). Patients in our sample were also more often married/cohabiting (36% vs. 27%, P = 0.02). In general, our sample appeared somewhat skewed towards having fewer young drug addicts compared with the national sample. Further information about sampling, subjects and methods has been described more extensively in previous papers from the index admission [2,27]. All patients gave their written informed consent to assessment at admission and to be contacted at follow-up.

Six years later (mean time = 75 ± 5 months), 33 patients (11%) had died and 21 deaths were substance related. Two died from cancer, one from suicide, and two for unknown reasons. The National Registry provided data for the deceased patients. Death certificates (cause of death) were obtained from the Cause of Death Registry (Division for Health Statistics in Statistics Norway). At baseline, the deceased patients (n = 33) were older (44.3 vs. 37.9 years, respectively, P = 0.002), more often alcoholics (73% vs. 52%, respectively, P = 0.024), and more likely to have a later onset of SUD (mean age at onset: 26.5 vs. 22.0 years, respectively, P = 0.024) than were the 254 surviving patients. In terms of lifetime Axis I disorders, the deceased patients had fewer social anxiety disorders (SADs) (23.3% vs. 48.6%, respectively, P = 0.009), major depression (25.0% vs. 46.4%, respectively, P = 0.022), and a lower number of Axis I disorders (2.7 ± 2.2 vs. 3.7 ± 2.6, respectively, P = 0.035) than did survivors. In terms of Axis II disorders, the deceased patients more often had schizoid (46.4% vs. 19.6%, respectively, P = 0.001) or dependent personality disorders (35.7% vs. 18.5%, respectively, P = 0.033) than did survivors.

The six-year follow-up questionnaire was mailed to the surviving individuals of the index admission sample (n = 254). Most of the patients were also contacted by telephone. Subjects were paid 300 Norwegian kroner (approximately 38 Euros) for completing the form. Among the surviving patients (n = 254), 63% (n = 160, 29% of whom were women, mean age = 45.1 ± 11.2 years) returned the questionnaire and these constituted the follow-up sample. Of the group that did not respond at follow-up (n = 94), 14 patients actively refused to participate, 23 were not located, and 57 subjects received the assessment form twice (limit set by the Norwegian Data Inspectorate), but did not reply to it. The participant rate was similar to that for former patients from both outpatient and inpatient facilities. Our follow-up sample (n = 160) did not differ significantly from those who did not participate (n = 94) with regard to sociodemographic or baseline variables: substance-use variables, Axis I and II disorders, levels of mental distress (HSCL-25), inpatient or outpatient status, and treatment history before admission.

At the six-year follow-up, not all members of the follow-up sample had completed the HSCL-25 in an acceptable way (21 subjects at baseline and two subjects at follow-up), leaving us with 137 patients (86% of the follow-up sample (137/160)) who had completed the HSCL-25 both at baseline and at follow-up. These 137 persons constitute the sample of the present study. There were no significant differences between these participants and the non-participants (n = 117), including both the non-responders (n = 94) and responders without HSCL-25 at T1 or T2 (n = 23) with regard to sociodemographic, substance use, or mental health variables.

### Evaluation at the index admission (baseline, T1)

The rating instrument used to assess sociodemographic and treatment history was the Norwegian National Client Assessment form.

Lifetime Axis I disorders were evaluated with a structured interview, the Composite International Diagnostic Interview (CIDI) [28]. Diagnosis of substance abuse, harmful use, and dependence was made with the CIDI (based on DSM-IV and ICD-10 criteria). ICD-10 diagnosis were used for the non-substance Axis I disorders. The CIDI has shown good feasibility in general populations and high inter-rater reliability, and has been subjected to tests of reliability and validity with satisfactory results [29]. This interview was also used to identify age of onset of SUD, i.e., the first year in which the person fulfilled the criteria for a SUD diagnosis. An early age of onset of SUD was defined as the onset of either substance abuse or substance dependence before the age of 18 years. The CIDI was also used to classify the patient's main substance of abuse as either alcohol dependence with no other drug dependence or abuse, or as poly-substance dependence. Among poly-substance dependent patients 85% used heroin and/or amphetamine as the primary substance of abuse.

Mental distress was measured with the self-report instrument, the Hopkins Symptom Checklist (HSCL-25) [30], which is widely used in both population studies [31,32] and patient populations [33]. The HSCL-25 consists of 25 items that predominantly measure anxiety and depression symptoms over the course of the previous week. The mean total sum score is called the General Symptom Index (GSI). In our sample, 143 patients had fulfilled the HSCL-25 form at admission. Four patients answered fewer than 16 questions and were therefore excluded from the analyses, whereas eight patients who answered between 21 and 24 questions were included. Cronbach's alpha was 0.94. There was a very high correlation between both the number of lifetime Axis I and Axis II disorders and the HSCL-25 score at baseline (r = 0.61, r = 0. 48, respectively).

Personality disorders were assessed using the self-report instrument the Millon's Clinical Multiaxial Inventory (MCMI-II) [34], a 175-item instrument to which patients respond "true" or "false". This instrument measures 13 personality scales according to the DSM-III-R diagnostic system. The cut-off value for "caseness" on the different MCMI-II scales is a base rate score of 85 or more. This is a more stringent criterion in assigning a suggested diagnosis than is recommended by the MCMI manual, but was used to ensure that the diagnostic criteria for any given personality disorder was satisfied. Findings from several studies of the psychometric properties of the MCMI-II have reported acceptable test-retest reliability, and generally acceptable levels of convergent and discriminant validity of the scales [35].

The self-report instruments were completed in association with the CIDI interview. Among inpatients, the CIDI interview and the self-report instruments were conducted four weeks after discontinuation of substance abuse at the earliest. This demand of four weeks of sobriety was difficult to achieve in all outpatients. Therefore, we decided to include those clients that failed to meet this criterion if they were sober in the assessment situation.

### Evaluation at the six-year follow-up (T2)

Because of a lack of resources, it was not possible to conduct personal interviews (CIDI) to evaluate psychiatric diagnoses and SUD at follow-up. As an alternative, well-established self-report instruments were used. The same self-report instrument used at baseline, the HSCL-25, was used at follow-up to measure mental distress. Cronbach's alpha was 0.95.

Two self-report instruments measured substance use both at the follow-up and during the preceding year: the Alcohol Use Disorders Identification Test (AUDIT) and the Drug Use Disorders Identification Test (DUDIT). These are instruments with good potential reliability and validity [36,37]. Both screening instruments are used to identify persons with a problematic use of substances during the previous 12 months. The instruments are standardized and based on selected criteria for substance abuse, harmful use, and dependence, according to the ICD-10 and DSM-IV diagnostic systems. AUDIT was developed from a six-country WHO collaborative project as a screening instrument for hazardous and harmful alcohol consumption [38]. It is a 10-item questionnaire that includes sections on alcohol consumption, alcohol dependence, and alcohol-related problems. Responses to each question are scored from 0 to 4, giving a maximum possible score of 40. Screening levels for hazardous alcohol use are ≥ 8 points for men and ≥ 6 points for women. In our study, reliability according to Cronbach's alpha was 0.94.

DUDIT was developed and tested in Sweden [37]. It is an 11-item self-report instrument intended for use in parallel with AUDIT. Screening levels for drug-related problems are ≥ 6 points for men and ≥ 2 for women out of a maximum of 44 points. Cronbach's alpha was 0.95.

Patients were classified into one of two groups, "abstainers" or "relapsers", based on their AUDIT and DUDIT scores. The "abstainers" (n = 41) consisted of persons with no drug- or alcohol-related problems at follow-up or during the immediately preceding 12 months (based on an AUDIT score of < 8 points for men and < 6 points for women and a DUDIT score of < 6 points for men and < 2 points for women). The "relapsers" (n = 96) consisted of persons with drug- or alcohol-related problems during the preceding year and at follow-up (based on an AUDIT score of ≥ 8 for men and ≥ 6 for women, or a DUDIT score of ≥ 6 for men and ≥ 2 for women).

### Statistics

Quantitative measures were compared among groups with analyses of variance (independent t-test, paired samples test, and one-way ANOVA) and with χ^2 ^tests. Bivariate correlations were measured using Pearson's product-moment correlation coefficient. In table 1, 2, 3 we have controlled for the level of HSCL-25 at T1 using linear regression analyses, corresponding to ANCOVAs with HSCL-25 at T2 as the dependent variable using the independent variables investigated as factors and HSCL-25 score at T1 as a linear covariate. Finally, hierarchical multiple regression analysis was used, with the HSCL-25 score at follow-up as the dependent variable. Four blocks of independent variables were used to explore the predictive value of each of the specific lifetime Axis I and II disorders (one by one) and also the number of these disorders on the level of mental distress at follow-up. Block one comprised the different individual Axis I disorders (one by one) or, separately the number of Axis I disorders (0–11). Block two comprised the different individual Axis II disorders (one by one) or, separately the number of Axis II disorders (0–9). When each of the different Axis I disorders was tested, block two consisted of the number of Axis II disorders, and when the different Axis II disorders were tested, block one consisted of the number of Axis I disorders. Block three was the baseline measures of sociodemographic variables (sex and age), the type of main substance of abuse at the index admission, and age at SUD onset; block four represented substance use at follow-up. HSCL-25 scores at T1 and T2 showed a moderate test-retest correlation (r = 0.43). In a separate analysis we wanted to explore whether HSCL-25 scores at T1 would affect the association between HSCL-25 scores at T2 and the other putative predictors included in the regression equation (table 4). In table 4 we reported only the first four blocks due to the high correlation between Axis I disorders and HSCL-25 at T1 (r = 0.61). When we control for HSCL-25 scores at T1 the effect of a given independent variable on HSCL-25 at T2 will reflect differential change in mental distress from T1 to T2. All analyses were conducted using SPSS for Windows, version 11.0 (SPSS 2005).

**Table 1:** Mean score and standard deviation for HSCL-25 at baseline and follow-up, according to sociodemographic variables at baseline and substance-use variables at both at baseline and follow-up. Paired t-test and independent t-test.

	**T1**		**T2**			
	**Mean**	**SD**	**Mean**	**SD**	**P**^**a**^	**P**^**b**^
Total sample (n = 137)	2.08	0.61	2.01	0.63	0.219	
Age						
< 30 years (n = 28)	2.12	0.63	2.00	0.61	0.371	0.933
≥ 30 years (n = 109)	2.07	0.61	2.01	0.64	0.366	
Sex						
Male (n = 97)	2.03	0.62	2.03	0.65	0.996	0.513
Female (n = 40)	2.19	0.58	1.96	0.58	0.019	
Education						
Only primary school (n = 59)	2.20	0.62	2.11	0.61	0.317	0.150
More than primary school (n = 66)	2.00	0.60	1.95	0.65	0.543	
Civil status						
Not married/cohabiting (n = 77)	2.13	0.66	2.03	0.63	0.200	0.650
Married/cohabiting (n = 50)	2.02	0.56	1.98	0.65	0.589	
Employment %						
Yes (n = 44)	2.04	0.61	2.03	0.66	0.940	0.911
No (n = 83)	2.13	0.62	2.02	0.62	0.098	
Main substance of abuse						
Alcohol (n = 77)	2.04	0.63	2.04	0.63	0.930	0.578
Poly-substance abuse (n = 60)	2.13	0.59	1.98	0.63	0.106	
Age at onset of substance use disorder						
≥ 18 years (n = 76)	2.07	0.63	1.90	0.64	0.022	0.020
< 18 years (n = 61)	2.09	0.58	2.15	0.59	0.489	
Use of substances at T2						
"Abstainers" (n = 41)	1.94	0.60	1.61	0.59	0.002	< 0.001
"Relapsers" (n = 96)	2.14	0.61	2.18	0.56	0.514	

^a^P value on paired t-test.

^b^P value on independent t-test T2

**Table 2:** Mean score and standard deviation for HSCL-25 at baseline and follow-up, according to Axis I disorders. Paired t-test and independent t-test.

	**T1**		**T2**			
	**Mean**	**SD**	**Mean**	**SD**	**P**^**a**^	**P**^**b**^
Any Axis I disorder						
Yes (123)	2.14	0.60	2.07	0.61	0.287	0.001
No (14)	1.60	0.42	1.49	0.53	0.445	
Affective disorders						
Yes (n = 85)	2.26	0.59	2.12	0.63	0.064	0.018
No (n = 50)	1.80	0.53	1.86	0.59	0.492	
Major depression						
Yes (n = 59)	2.27	0.61	2.15	0.61	0.177	0.44
No (n = 76)	1.95	0.58	1.93	0.63	0.802	
Dysthymia						
Yes (n = 50)	2.28	0.55	2.17	0.70	0.228	0.021
No (n = 83)	1.95	0.62	1.91	0.57	0.573	
All anxiety disorders						
Yes (n = 110)	2.18	0.61	2.10	0.63	0.267	< 0.001
No (n = 27)	1.69	0.43	1.64	0.49	0.583	
Agoraphobia^c^						
Yes (n = 61)	2.33	0.57	2.20	0.63	0.178	0.001
No (n = 74)	1.87	0.57	1.86	0.60	0.816	
Social anxiety disorder						
Yes (n = 65)	2.34	0.60	2.24	0.60	0.286	< 0.001
No (n = 69)	1.86	0.52	1.80	0.59	0.485	
Simple phobias						
Yes (n = 60)	2.29	0.69	2.08	0.65	0.034	0.268
No (n = 76)	1.92	0.48	1.96	0.61	0.508	
Generalized anxiety disorder						
Yes (n = 25)	2.32	0.62	2.27	0.67	0.701	0.022
No (n = 112)	2.03	0.60	1.95	0.61	0.243	
Post-traumatic stress disorder						
Yes (n = 29)	2.35	0.66	2.07	0.57	0.035	0.558
No (n = 107)	2.01	0.58	1.99	0.65	0.739	
Somatization disorder						
Yes (n = 39)	2.35	0.60	2.21	0.62	0.291	0.022
No (n = 97)	1.97	0.59	1.94	0.62	0.558	
Number of Axis I disorders						
0 (n = 14)	1.60	0.42	1.49	0.53	0.445	< 0.001^d^
1–3 (n = 58)	1.85	0.48	1.95	0.58	0.167	
4 + (n = 65)	2.39	0.59	2.17	0.63	0.021	

^a^P value on paired t-test.

^b^P value on independent t-test HSCL-25 T2.

^c^Agoraphobia with and without panic attack.

^d^One-way ANOVA: number of Axis I disorders, df = 11, F = 3.5, P < 0.001.

**Table 3:** Mean score and standard deviation for HSCL-25 at baseline and follow-up, according to Axis II disorders. Paired t-test and independent t-test.

	**T1**		**T2**			
**Axis II disorders**	**Mean**	**SD**	**Mean**	**SD**	**P**^**a**^	**P**^**b**^
Any Axis II disorder						
Yes (98)	2.22	0.60	2.10	0.65	0.090	0.008
No (36)	1.76	0.51	1.79	0.52	0.733	
Paranoid						
Yes (n = 6)	2.40	0.56	2.55	0.31	0.638	0.035
No (n = 128)	2.08	0.61	1.99	0.63	0.139	
Schizoid						
Yes (n = 23)	2.45	0.65	2.25	0.54	0.224	0.049
No (n = 111)	2.02	0.58	1.97	0.64	0.397	
Schizotyp						
Yes (n = 17)	2.66	0.58	2.34	0.62	0.088	0.025
No (n = 117)	2.01	0.57	1.97	0.62	0.496	
Antisocial						
Yes (n = 43)	2.17	0.60	2.14	0.65	0.728	0.136
No (n = 91)	2.06	0.61	1.96	0.62	0.161	
Borderline						
Yes (n = 43)	2.45	0.58	2.26	0.61	0.110	0.002
No (n = 91)	1.92	0.55	1.90	0.62	0.699	
Histrionic						
Yes (n = 17)	2.14	0.61	2.04	0.83	0.533	0.849
No (n = 117)	2.09	0.61	2.01	0.60	0.238	
Narcissistic						
Yes (n = 19)	2.14	0.60	2.21	0.76	0.653	0.161
No (n = 115)	2.08	0.61	1.99	0.61	0.117	
Avoidant						
Yes (n = 52)	2.45	0.57	2.28	0.58	0.098	< 0.001
No (n = 82)	1.87	0.52	1.85	0.61	0.802	
Dependent						
Yes (n = 24)	2.43	0.62	2.19	0.69	0.051	0.129
No (n = 110)	2.02	0.58	1.98	0.62	0.529	
Compulsive						
Yes (n = 8)	2.34	0.86	2.19	0.77	0.544	0.420
No (n = 126)	2.08	0.59	2.01	0.63	0.226	
Passive-aggressive						
Yes (n = 54)	2.32	0.57	2.28	0.60	0.594	< 0.001
No (n = 80)	1.94	0.59	1.84	0.60	0.207	
Self-defeating						
Yes (n = 36)	2.59	0.59	2.33	0.62	0.045	< 0.001
No (n = 98)	1.91	0.51	1.90	0.60	0.886	
Aggressive-sadistic						
Yes (n = 33)	2.22	0.54	2.23	0.59	0.291	0.029
No (n = 101)	2.05	0.62	1.95	0.64	0.558	
Number of Axis II disorders						
0 (n = 36)	1.76	0.51	1.79	0.52	0.733	< 0.001^c^
1–3 (n = 47)	1.99	0.52	1.82	0.64	0.083	
4 + (n = 51)	2.43	0.59	2.36	0.56	0.485	

^a^P value on paired t-test.

^b^P value on independent t-test HSCL-25 T2

^c^One-way ANOVA: number of Axis II disorders, df = 9, F = 4.0, P < 0.001.

**Table 4:** Hierarchical multiple regression with the HSCL-25 score at follow-up as the dependent variable and four blocks of independent variables. Standardized regression coefficients and adjusted R squares for each block (n = 134).

	**B**	**SE**	**Standardizert B**	**Adjusted R**^**2**^
Block 1				0.13
Number of Axis I disorders	0.090	0.019	0.374***	
Block 2				0.19
Number of Axis I disorders	0.063	0.021	0.260**	
Number of Axis II disorders	0.068	0.021	0.282**	
Block 3				0.24
Number of Axis I disorders	0.066	0.021	0. 274**	
Number of Axis II disorders	0.066	0.021	0.273**	
Sex (male = 1)	0.079	0.111	0.057	
Age	-0.004	0.005	-0.062	
Main substance of abuse (alcohol = 1)	0.349	0.122	0.274**	
Onset SUD (below 18 years = 1)	0.300	0.118	0.236*	
Block 4				0.33
Number of Axis I disorders	0.049	0.020	0.203*	
Number of Axis II disorders	0.068	0.019	0.280**	
Sex (male = 1)	0.054	0.105	0.039	
Age	0. 000	0.005	-0. 007	
Main substance of abuse (alcohol = 1)	0.288	0.116	0.226*	
Onset of SUD (below 18 years = 1)	0.238	0.113	0.187*	
Use of substances at T2 ("relapser" = 1)	0.431	0.104	0.312***	

*P < 0.05

**P < 0.01

***P < 0.001

### Ethics

The study protocol was reviewed both at baseline and at follow-up and approved by the Regional Committee for Medical Research Ethics and by the Norwegian Data Inspectorate.

## Results

### Level of and change in mental distress at the six-year follow-up

As shown in Table 1 there was a significant difference in the **level **of mental distress at T2 between the age at onset of SUD groups. The late-onset group had a lower level of mental distress compared with that of the early-onset group (1.90 ± 0.64 vs. 2.15 ± 0.59, respectively, P = 0.020). At T2, the "abstainers" had significantly lower mental distress than that of the "relapsers" (1.61 ± 0.59 vs. 2.18 ± 0.56, respectively, P < 0.001).

On a group level, there was no significant difference in mental distress between T1 (index admission) and T2 (follow-up) (2.08 ± 0.61 vs. 2.01 ± 0.63, respectively, P = 0.219). Concerning the course in different subgroups, Table 1 shows that among women, but not among men, there was a significant decrease in mental distress (2.19 ± 0.58 vs. 1.96 ± 0.58, P = 0.019). This was also the case among patients with late-onset SUD, with mental distress decreasing from 2.07 ± 0.63 to 1.90 ± 0.64 (P = 0.022). In patients who were alcohol-dependent at admission, mental distress was exactly the same at T1 and T2 (2.04 ± 0.63, P = 0.930), whereas in patients who were poly-substance dependent at admission, mental distress tended to decrease but not significantly (from 2.13 ± 0.59 to 1.98 ± 0.63, P = 0.106).

Persons who were classified as "abstainers" at follow-up showed a decrease in mental distress (1.94 ± 0.60 vs. 1.61 ± 0.59, P = 0.002), whereas the "relapsers" reported a stable HSCL-25 level (2.14 ± 0.61 at T1 vs. 2.18 ± 0.56 at T2, P = 0.514). Women "abstainers" displayed a significant decrease in mental distress (from 2.14 ± 0.63 to 1.41 ± 0.37, P < 0.001), whereas men did not (from 1.85 ± 0.57 to 1.69 ± 0.66, P = 0.174) (not shown in Table 1). When controlling for HSCL-25 at T1 in the analysis in table 1 age at onset of substance use disorder (P = 0.013) and use of substances at T2 (P = < 0.001) still remained significant.

The level of mental distress at T2 was significantly higher for patients with any lifetime Axis I disorder (2.07 ± 0.61 vs. 1.49 ± 0.53, P = 0.001) compared with that of patients with no Axis I disorder. This was the case also for nearly all the specific Axis I disorders, except major depression, simple phobias, and post-traumatic stress disorder (PTSD). Patients with ≥ 4 lifetime Axis I disorders assessed at baseline showed a significant decrease in HSCL-25 scores from baseline to follow up (2.39 ± 0.59 vs. 2.17 ± 0.63, P = 0.021), but the level was still high. Looking at the change in mental distress in each single Axis I disorder there was a significant decrease in patients only in two diagnostic groups: simple phobias (2.29 ± 0.69 vs. 2.08 ± 0.65, P = 0.034) and PTSD (2.35 ± 0.66 vs. 2.07 ± 0.57, P = 0.035).

The level of mental distress at T2 was significantly higher for patients with any Axis II disorder (2.10 ± 0.65 vs. 1.79 ± 0.52 in patients with no disorder, P = 0.008), and in those with eight of the 13 personality disorders.

Patients with no personality disorder reported a stable and relatively low level of mental distress (from 1.76 ± 0.51 to 1.79 ± 0.52, P = 0.733), whereas patients with ≥ 4 personality disorders reported a stable but high level of mental distress (from 2.43 ± 0.59 to 2.36 ± 0.56, P = 0.485). Concerning each specific personality disorder (Table 3), there was only a slight significant decrease in mental distress in those patients with a self-defeating personality disorder (2.59 ± 0.59 vs. 2.33 ± 0.62, P = 0.045).

There was no significant decrease in mental distress in patients who had been treated in the substance abuse field (68%) or in the mental health care system (54%) between T1 and T2. In all, 79% were treated either in one or two of the treatment systems (not shown in Table 3).

In table 2 any Axis I disorder (P = 0.028), all anxiety disorder (P = 0.034) and social anxiety disorder (P = 0.017) remained significant after controlling for baseline mental distress. In table 3 only number of Axis II disorders (P = 0.008) and passive-aggressive disorder (P = 0.006) remained significant.

To explore whether Axis I or II disorders predicted the level of mental distress at follow-up, we used a hierarchical multiple regression analysis (Table 4), with the HSCL-25 score at follow up as the dependent variable. The independent variables were each of the different Axis I and II disorders, one by one (1 = yes and 0 = no), or the number of Axis I and II disorders, age, and sex (1 = male and 0 = female), onset of SUD (1 = below 18 years and 0 = 18 years or older), main substance of abuse (1 = alcohol dependence and 0 = poly-substance dependence) and use of substances at T2 (1 = "relapser" and 0 = "abstainer"). The number of Axis I and II disorders, main substance of abuse, and onset of SUD were significantly and independently related to a higher level of HSCL-25 at T2, whereas sex and age were not (R^2 ^= 0.24). These findings were, in principle, the same when we controlled for "relapsers"/"abstainers" at T2. The overall model was significant (F [7,126] = 10.21, P < 0.001). When we looked at single Axis I disorders, SAD, generalized anxiety disorder (GAD), and somatization disorder were significantly and independently related to higher mental distress at T2 controlled for Axis II disorders. However, the standardized beta (0.203) and adjusted R^2 ^(0.327) values were slightly higher for the number of Axis I disorders compared with those for each of the specific disorders. Among these disorders, SAD had the highest standardized beta (0.195) and adjusted R^2 ^(0.326) values compared with those for GAD and somatization disorder.

In the same types of analyses, we found that having any Axis II disorder (controlled for Axis I disorders) implied a significantly higher level of mental distress at follow-up. Concerning single personality disorders, five disorders were significantly and independently related to a higher level of mental distress at T2: borderline, passive-aggressive, aggressive-sadistic, avoidant, and self-defeating. However, as regarding Axis I disorders, the number of Axis II disorders was a better predictor than each of the specific Axis II disorders. When controlling for treatment (in all) between T1 and T2 (measured by self-report), the only change in predictors was that age of onset of SUD became non-significant (90% of patients in the early-onset SUD group had been treated between T1 and T2).

HSCL-25 at T1 was a significant predictor of HSCL-25 score at T2 (zero-order correlation corresponding to a "stability coefficient" or "test-retest" correlation). Either number of Axis I disorders, or the specific Axis I disorders were significant predictors of HSCL-25 at T2 when HSCL-25 scores at T1 were entered into the equation. The other predictors remained significant even when controlling for baseline mental distress.

## Discussion

### The course of mental distress

The level of mental distress at follow-up was as high as that at admission (2.01 ± 0.63 vs. 2.08 ± 0.61, respectively, P = 0.219), and far above the level in the general population. In a population study from Norway [31] using the HSCL-25, the mean score was 1.33 (SD:SE = 0.004). The stability of mental distress over six years is supported by some of the literature [13-16], and shows that, on a group level among substance-dependent subjects who seek treatment, the level of mental distress will not go away by itself or with time. The level of mental distress in our sample was also high at both baseline and follow-up, even when compared with other follow-up studies of SUD patients [17,39]. We also found more Axis I disorders at baseline than did other studies, like ours, that used CIDI [4,40,41].

In the different subgroups, the most important finding was that mental distress substantially decreased in the "abstinent" group, especially among women, whereas it remained stable and high in the "relapse" group. The mean score at follow up among the "abstinent" women (1.41 ± 0.37) was similar to the level of mental distress in the general population study [31], where the mean level for women was 1.37 (SD:SE = 0.006). Among "abstinent" men (1.69 ± 0.66) mental distress was higher than for men in the population study1.28 (SD:SE = 0.006). Therefore, our group of abstinent women seems to have obtained a distribution of mental distress that is very similar to that of women in the general population, whereas the abstinent group of men still has a somewhat higher level of distress than that of men in the general population.

Our findings may also indicate that mental distress in women is more strongly associated with ongoing addictive behaviour than it is among men. In a 16-month follow-up study, Tomasson and Vaglum found a significant relationship between duration of sobriety and lower psychiatric distress, but this association was less clear for women [23]. In our multivariate analyses, sex was not an independent predictor of the level of mental distress at T2.

In all, it is important to remember that all studies, including our own, show that eliminating the addictive behaviour does not always lead to a clinically significant reduction in mental distress. Consequently, Axis I and II disorders, and not only the SUD, should be addressed in treatment programmes.

Among single Axis I disorders, only patients with simple phobias and PTSD had a significant decrease in mental distress. In our study, as in others [42], simple phobias were not as severe as many of the other Axis I disorders and are therefore possibly easier to eradicate. The decrease in mental distress we observed in patients with PTSD is in contrast to a review of SUD patients with PTSD [43] that reported poor outcomes on mental distress and that the negative effect of a comorbid PTSD diagnosis was greater than the effects of other comorbid psychiatric disorders. However, the generalizability of the studies included in that review can be discussed with regard to both sampling (male SUD patients in VA treatment centres) and methodology (lack of structured clinical interview). When controlling for baseline mental distress only a few of the differences in table 2 and 3 remained significant, primarily the variables with the greatest differences in HSCL-25 score at T2. Much of the impact of the various Axis I and II disorders on HSCL-25 at T2 was due to the HSCL-25 level at index admission.

### Predictors of mental distress at follow-up

The number of Axis I disorders was significant and independently related to the level of mental distress when we controlled for age, sex, Axis II disorders, and substance-use variables at both T1 and T2. This is in accordance with the results of several studies with shorter follow-up interval [20-24]. Only patients with ≥ 4 Axis I disorders at baseline reported a significant decrease in their levels of mental distress from baseline to follow up. Nevertheless, the level of mental distress was still very high in this subgroup. Grella et al. [6], Melberg et al. [39], and Liskow et al. [44] also found the greatest reduction in psychiatric severity in those groups who were highest at baseline. This may be due to the fact that patients seek help when their mental distress is at a maximum.

In the multivariate analyses, anxiety disorders (GAD, SAD, sum of anxiety disorders) together with somatization disorders were significant and independently related to a high level of mental distress at follow-up. Haver and Gjestad [45] also found phobic anxiety to be an important predictor of outcome in female alcohol-dependent patients, partly due to the influence phobic anxiety had on depression. In general, anxiety disorders are more stable than affective disorders [46], and they should always be the focus of therapeutic interventions among SUD patients. Compared with the other Axis I disorders that had a significant and independent impact on mental distress at follow-up, social anxiety disorder was a more important predictor of mental distress. There is a high degree of impairment associated with SAD and higher risk of persistence compared with that of other anxiety disorders [46,47]. Patients with SAD in our index sample experienced more comorbid mental disorders than did patients without SAD [27], and most patients with SAD in our sample exhibited generalized SAD, a subtype that causes more impairment than the non-generalized type [48].

No single affective disorder had an impact on the level of mental distress at follow-up, but because the total number of Axis I disorders had such an impact, affective disorders may have made a more non-specific contribution. The literature concerning SUD patients with depression confirms our finding that psychiatric symptoms may improve when sobriety occurs. Hatsukami and Pickens [49] and Driessen et al. [50] also reported that the rate and severity of depressive symptoms among sober alcohol-dependent patients were similar to those of the general population. However, those studies were short-term follow-up studies. In a 10-year follow-up study of patients with major depressive disorders with and without alcohol dependence, there was a twofold greater likelihood of improvement in the major depressive disorders in patients without a current alcohol disorder compared with that of patients with an active alcohol disorder [51]. In a five-year follow-up study of alcohol-dependent patients with major depression, remission of alcoholism strongly and significantly increased the chances of remission of depression [52]. Hodgins et al. followed alcohol-dependent patients for three years and found that among patients with good drinking outcomes, 26% suffered depression compared with 60% of those with poor drinking outcomes [18].

In the multivariate analyses none of the Axis I disorders or number of Axis I disorders was a predictor of mental distress at T2 when controlling for HSCL-25 at T1. Much of the impact of Axis I disorders was due to shared variance with HSCL-25. HSCL-25 is a dimensional measure of anxiety and depressive symptoms whereas diagnosing Axis I disorders is a categorical way of measuring mostly anxiety disorders and affective disorders, i.e. closely related phenomena. Thus, there was a high correlation between Axis I disorders and HSCL-25, not least between a tally of the number of Axis I disorders and HSCL-25 scores (r = 0.61).

Both number of personality disorders and some specific disorders, mostly of the anxious and dramatic types, were independently related to a relatively poor outcome concerning mental distress, when we also controlled for sex, age, number of Axis I disorders, and substance use both at baseline and at follow-up. Those with a personality disorder continued to have a high level of mental distress from T1 to T2. The persistence of mental distress in patients with many Axis II disorders is consistent with a recent review showing that Axis II disorders are often associated with poor outcomes for psychiatric problems among SUD patients [25]. This underlines once more the importance of considering the personality disorders of SUD patients. When entering HSCL-25 at T1 into the analysis, the number of PD's still remained a significant predictor of mental distress at T2. This underscores the long-term impact of personality disorders on mental distress. Number of disorders may be a more robust measure than each specific disorder. Among the specific Axis II disorders only passive-aggressive still remained significant.

Our findings in this study demonstrate the importance of anxiety disorders for the level of mental distress at the six-year follow-up, whereas another study of the same follow-up sample [53] has shown that major depression and agoraphobia are significant and independent predictors of a harmful use of substances in the year prior to follow up. Together, these findings underscore the need to offer substance-dependent patients treatment that focuses not only on their substance abuse, but also on their anxiety, affective, and personality disorders.

On bivariate analysis, there was no significant difference in the mental distress of alcohol-dependent and poly-substance-dependent patients. However, in the multivariate analysis, the main substance of abuse (alcohol = 1) became a significant predictor of a high level of mental distress at T2 when we controlled for age or age at onset of SUD. Alcohol-dependent patients were older than poly-substance-dependent patients and they were older at the onset of SUD. In patients with an onset of SUD before 18 years, there was a significantly higher level of mental distress at T2 among alcohol-dependent patients than among poly-substance-dependent patients (2.39 ± 0.43 vs. 2.06 ± 0.43, respectively, P = 0.051). Even if poly-substance-dependent patients have more Axis I and II disorders [2], the level of mental distress in purely alcohol-dependent patients should not be underestimated.

The subgroup with an early age at onset of SUD had a significantly higher level of mental distress at follow-up (when we controlled for all other variables) compared with that of the late-onset group. At baseline the EO group were younger (33.1 vs. 42.9 years, P = < 0.001), more frequently poly-substance dependent (72% vs. 21%, P = < 0.001), had more often only primary school (62% vs. 35%, P = 0.002) and more often any Axis I disorder (97% vs. 84%, P = 0.016). The high level of mental distress in the EO group is in accordance with the finding of Babor et al., who reported higher average distress in type B alcoholics (with early onset of alcoholism) than that in type A alcoholics (with late-onset of alcoholism), in both one-year and three-year follow-up studies [54]. It seems that patients with an early onset of SUD are more seriously disturbed and less integrated into society.

In contrast to the majority of studies, we did find a direct, independent association between substance-use variables at admission and the level of mental distress at follow-up when we controlled for sex, age, Axis I and II variables, and for substance use during the year preceding follow-up. It is also important to note that, when we controlled for substance use at follow-up (and in the previous year), both Axis I and II disorders and baseline SUD variables remained independent and significant predictors of mental distress at follow-up. This shows that the impact of Axis I and II variables on the course of mental distress does not totally act through the substance use in the follow-up period, but that each of the variables had an independent impact on the mental distress outcome. This implies that one cannot expect total improvement in the other, potentially very disturbing, mental disorders, by limiting treatment to the addictive behaviour. The substance abuse field should involve the diagnosis and treatment of Axis I and II disorders as disorders independent of SUD.

### Limitations

A major strength of this study is that the sample is a consecutive sample, with heterogeneity of clients with regard to the number of facilities involved, the numbers of inpatients and outpatients, and the main substance of abuse. We have also used both a well-established structured interview (CIDI) and self-report instruments (MCMI, HSCL-25). The relatively long follow-up period (75 months) is also a strength, minimizing the "baseline effect" [22].

The representativeness of the sample is always a problem in clinical studies. The follow-up sample comprised only 20% of the patients that fulfilled the original inclusion criteria (137/690); 48% of those were included at index admission (137/287); and 54% of those from the index admission sample that were still living six years later (137/254). Our baseline sample was only somewhat skewed towards having fewer young poly-substance-dependent subjects compared with a national sample [26], but age has not been an important variable in this part of our study. In this follow-up study, we found no significant differences between dropouts and participants, either at baseline or in the follow-up sample, in terms of any of the central substance use or psychopathology variables. Nevertheless, a proportion of patients with high mental distress may have found it difficult to participate in the follow-up study, and we may have underestimated the level of mental distress at follow-up.

Although we assessed the most relevant substance use and psychopathology variables at baseline and at follow-up, other factors concerning substance use, such as important life events, treatment received, and social stability factors, may have influenced the outcome during the follow-up period. Furthermore, we were unable to examine either the stability of substance use or temporal changes in substance use. To evaluate mental distress at follow-up, a new CIDI interview would have been ideal, but due to a lack of resources, we had to use well-established self-report instruments with well-demonstrated sensitivity and specificity in measuring mental distress and in identifying harmful use of substances.

Another weakness of this study is that we did not have any confirmatory information regarding either the patients' use of substances nor mental health variables, but had to rely on the patients' willingness to report the truth. However, many recent studies have supported the validity of self-reports of substance use [55].

In all, although the external validity of the level of mental distress after six years may be unclear, we believe that the final sample we have followed up is well suited to identifying important predictors of mental distress among substance-abusing patients.

## Conclusion

In conclusion, this study shows that the level of mental distress among treatment-seeking SUD patients, on a group level, remained high after six years, but was significantly lower among those who had been abstainers, especially among women abstainers. Both the number of and the type of specific lifetime Axis I disorders (social anxiety disorder, GAD, and somatisation disorder), the number of and specific types of Axis II disorders (anxious and impulsive personality disorders), and the severity of SUD at the index admission were all independent predictors of a high level of mental distress at follow-up, even when we controlled for substance use at follow-up. These findings underscore the importance of the parallel diagnosis and treatment of both SUD and non-SUD Axis I and Axis II disorders in the same programme.

## Competing interests

The author(s) declare that they have no competing interests.

## Authors' contributions

KB undertook the conception and design of the study, the data collection, data analysis, and the interpretation of the data, and drafted the manuscript. AL undertook the conception and design of the study, the data collection, data analysis, and the interpretation of the data, and has been involved in revising the manuscript and has given final approval of the version to be published. PV contributed to the conception and design of the study, to the drafting and revision of the manuscript, and has given final approval of the version to be published. All authors have read and approved the final manuscript.

## Pre-publication history

The pre-publication history for this paper can be accessed here:


